# A Case of Chronic Right Hip Pain Revealing an Insidious, Incidental Eight-Centimeter Internal Aneurysm Among Multiple Diffuse Aneurysms

**DOI:** 10.7759/cureus.12709

**Published:** 2021-01-14

**Authors:** Candace Griffith, Riddhi Chaudhari, Rajesh Thirumaran

**Affiliations:** 1 Internal Medicine, Mercy Catholic Medical Center, Darby, USA; 2 Hematology/Oncology, Mercy Catholic Medical Center, Darby, USA

**Keywords:** internal iliac artery aneurysm, diffuse aneurysms, advanced destructive emphysema

## Abstract

The prevalence of internal iliac artery aneurysms (IIAA) is very low. Existing data on IIAA are scarce and mainly based on case reports and small retrospective series. We present the case of a 55-year-old African American man with a past medical history of HIV, hypertension, pulmonary embolism (PE), chronic obstructive pulmonary disease (COPD), coronary artery disease, polysubstance abuse, schizophrenia, depression, and bipolar disorder who presented to the emergency department with dyspnea on exertion. He was admitted for COPD exacerbation. He reported concerns of ambulatory chronic right hip pain, for which he underwent a CT, which revealed the presence of a partially visible right IIAA. A CT of his abdomen/pelvis revealed multiple aneurysms, including a partially thrombosed 8-cm fusiform right IIAA. Due to the presence of multiple aneurysms, the vascular surgery team was consulted, and elective repair was recommended. IIAA should be considered in the differential diagnosis of patients with significant smoking history and hip pain and acted upon immediately.

## Introduction

Internal iliac artery aneurysms (IIAA) are a rare condition with an estimated prevalence of 0.03%, representing 0.3% of all aortoiliac aneurysms; 70% of iliac aneurysms occur in the common iliac artery, and only 20% and 10% occur in the internal and external iliac arteries respectively [1]. In this report, we present the case of a patient with exacerbated chronic obstructive pulmonary disease (COPD) with subsequent incidental findings of multiple diffuse aneurysms, including a rare, partially thrombosed 8-cm IIAA.

## Case presentation

A 55-year-old African American man reported to the emergency department (ED) with concerns of exertional dyspnea. He was in respiratory distress and demonstrated labored breathing. His past medical history included HIV, hypertension, pulmonary embolism (PE), COPD, coronary artery disease, polysubstance abuse, schizophrenia, bipolar disease, and depression. Socially, the patient reported drinking alcohol daily, smoking cigarettes daily (10 cigarettes per day for 25 years), along with daily marijuana and cocaine use. His family history was pertinent for paternal lung cancer, maternal cerebrovascular accident, and heart disease in his sister. His vitals on presentation were as follows: blood pressure of 137/93 mmHg, heart rate of 100 beats/minute, respiratory rate of 22 breaths/minute, a body temperature of 97.9 °F, and pain level of 0/10; his oxygen saturation was 94% on room air and increased to 96% on 2 L of supplemental oxygen. The patient described one day of exertional shortness of breath, unrelieved by inhalers, and associated with a nonproductive cough. He admitted to recent drug use and medical non-compliance, including but not limited to his anticoagulation medication. A review of systems was significant for atraumatic ambulatory chronic right hip pain, which worsened with hip flexion.

**Figure 1:**
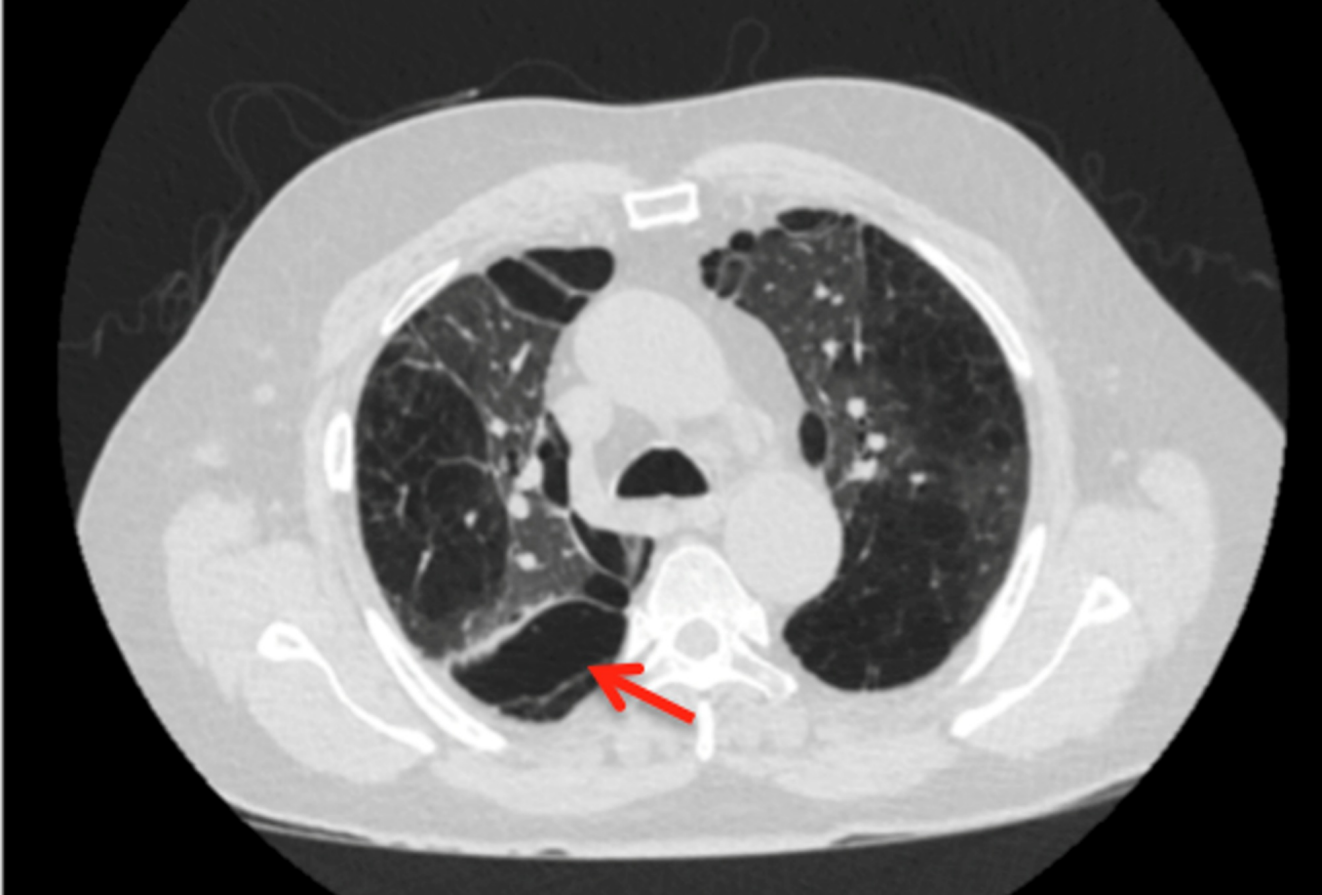
Advanced destructive emphysema (arrow)

**Figure 2:**
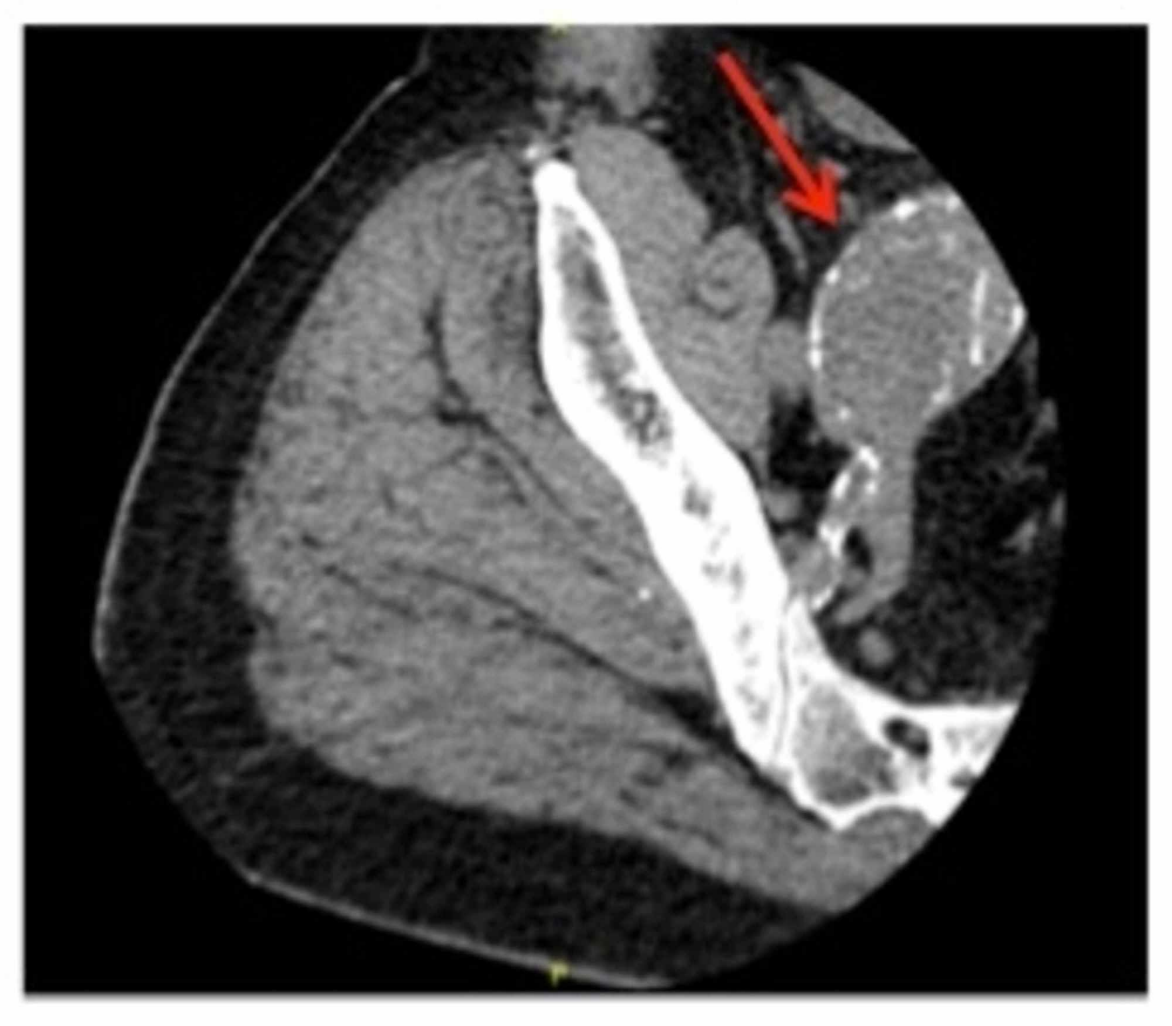
Partially visualized 4.4-cm mass in direct continuity with the internal iliac artery (arrow)

In the ED, he received three rounds of DuoNeb® (ipratropium bromide and albuterol sulfate) inhalation solution, 125 mg intravenous methylprednisolone, and 25 mg subcutaneous terbutaline. His chest X-ray showed no active pulmonary disease. He was admitted for COPD exacerbation, and a CT angiography of the chest ruled out a new PE but showed advanced destructive emphysema (Figure 1). A CT of the right hip (Figure 2) revealed no fracture but incidentally showed an abnormality that warranted a CT of the abdomen and pelvis.

**Figure 3:**
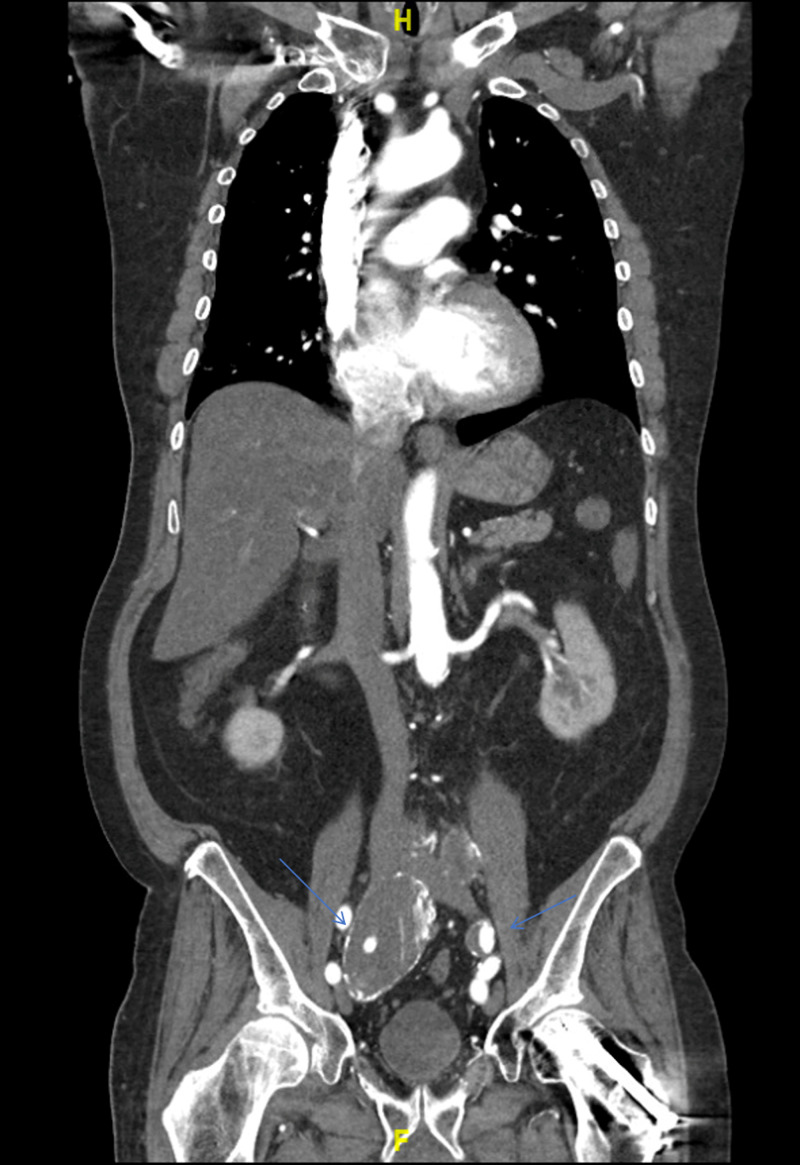
Fusiform aneurysm (5.4 x 5.2 x 8 cm) of the right internal artery, left common iliac artery (2.9 x 2.6 cm)

**Figure 4:**
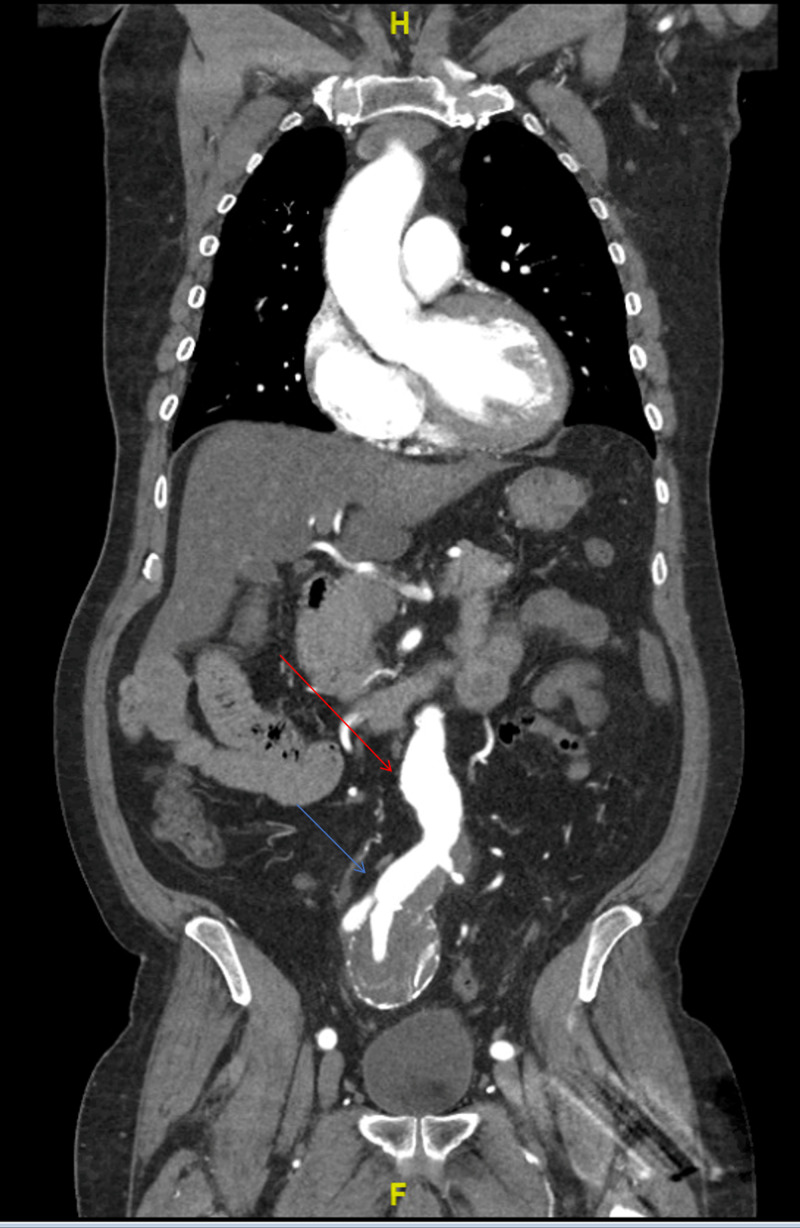
Fusiform aneurysm centered around the right internal iliac artery (blue arrow, 5.4 x 5.2 x 8 cm), which extends around the right common artery. The aneurysm is peripherally calcified and contains a partially calcified thrombus. Fusiform infrarenal aortic aneurysm (red arrow, 3.4 cm)

**Figure 5:**
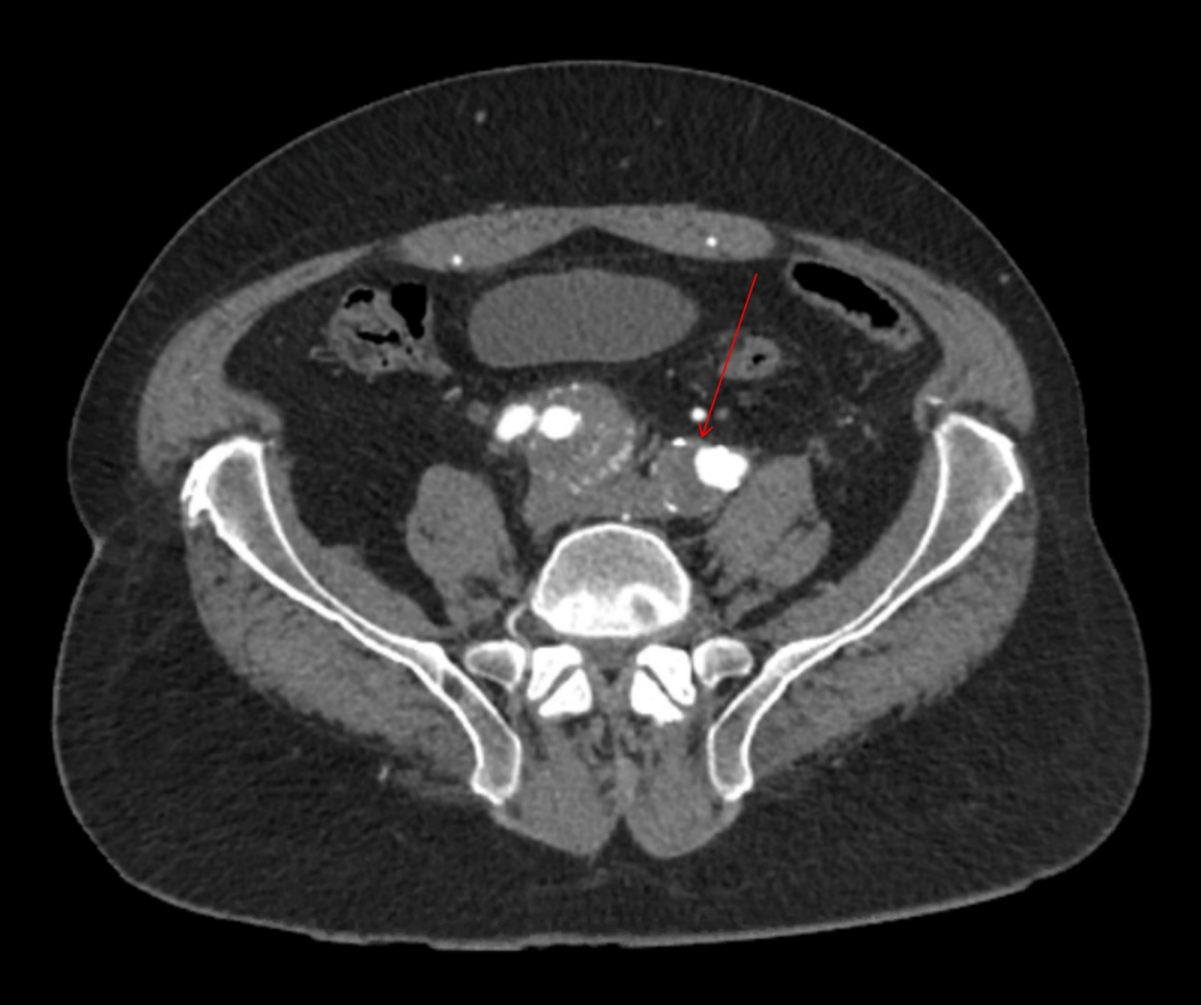
Left common iliac artery (2.9 x 2.6 cm) Note that bifurcation of the artery has not occurred on the left side, where both left internal and external iliac arteries are visible

**Figure 6:**
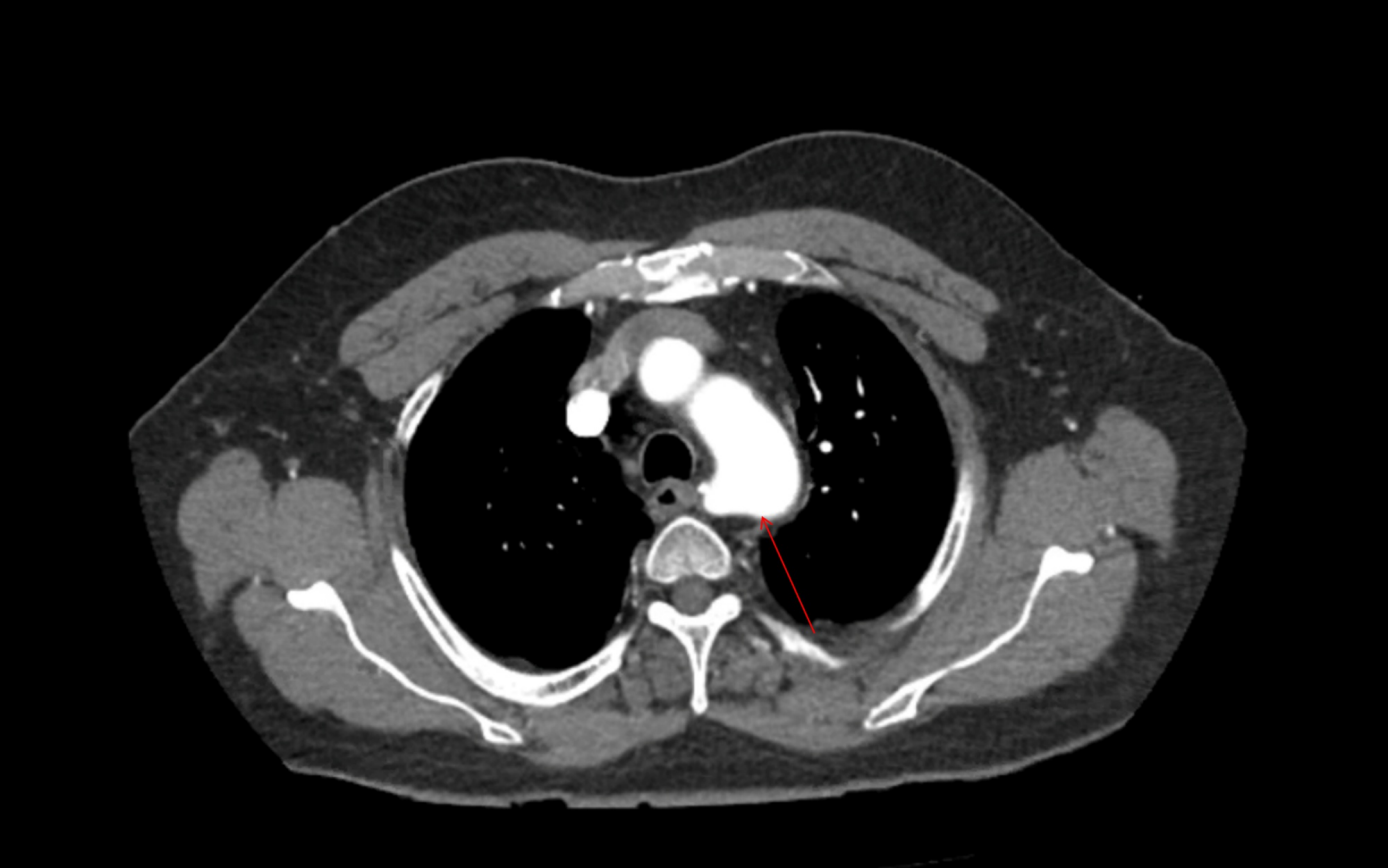
Focal aneurysmal outpouching (1.4 x 1.3 cm) at the medial aspect of the aortic arch, from where the right vertebral artery originates

The CT of the abdomen and pelvis revealed a partially thrombosed 8-cm fusiform right IIAA (centered around the right internal iliac artery, which extends around the right common artery), a 2.9-cm left common iliac artery aneurysm, a 3.4-cm fusiform infrarenal abdominal aortic aneurysm, and a 1.4-cm focal aneurysmal outpouching of the aortic arch (Figure 3, Figure 4, Figure 5, Figure 6).

## Discussion

An aneurysm is a segmental, full-thickness dilation of a blood vessel, 50% greater than its normal diameter. Normal limits vary with age, gender, and body habitus. True aneurysm formation is due to a loss of mechanical integrity of the vessel wall resulting from an altered balance between the production and degradation of the vascular wall constituents. The etiology of the imbalance is multifactorial (likely inflammatory, immunologic, mechanical, and/or genetic). Inflammatory diseases could be Behçet's disease, Kawasaki disease, and/or syphilis. Incidentally, our patient’s rapid plasma reagin result was negative. A false aneurysm (pseudoaneurysm) is a result of a breach in the vessel wall resulting in blood leaking out but contained by adventitia.

Although the dimensions that define an aneurysm are dependent on the sex of the patient and portion of the artery involved, a common iliac artery (Figure 3) that is greater than 1.7 cm in men or 1.5 cm in women is considered ectatic, and a diameter greater than 2.5 cm is considered aneurysmal [2]. An ectatic aorta should be closely monitored and followed up by ultrasound imaging.

Risk factors associated with IIAA include smoking, male sex, advancing age, Caucasian descent, atherosclerosis, positive family history, connective tissue disorders (Marfan syndrome or Ehlers-Danlos syndrome), and prior aortic surgery or instrumentation. The role of smoking and activated neutrophil elastase activity (and perhaps a more subtle defect in elastin) has been argued to be a provoking factor of aneurysm formation in patients with concomitant obstructive lung disease for developing multiple diffuse aortic aneurysms, as seen in our patient [3]. 

Although data on the natural history of IIAAs are limited due to their rare occurrence, expansive growth and subsequent rupture of aneurysms have been well documented. According to one of the largest series on ruptured IIAA published in the Journal of Vascular Surgery in January 2017, the mean diameter of aneurysms at the time of rupture was almost 7 cm [4].

**Figure 7:**
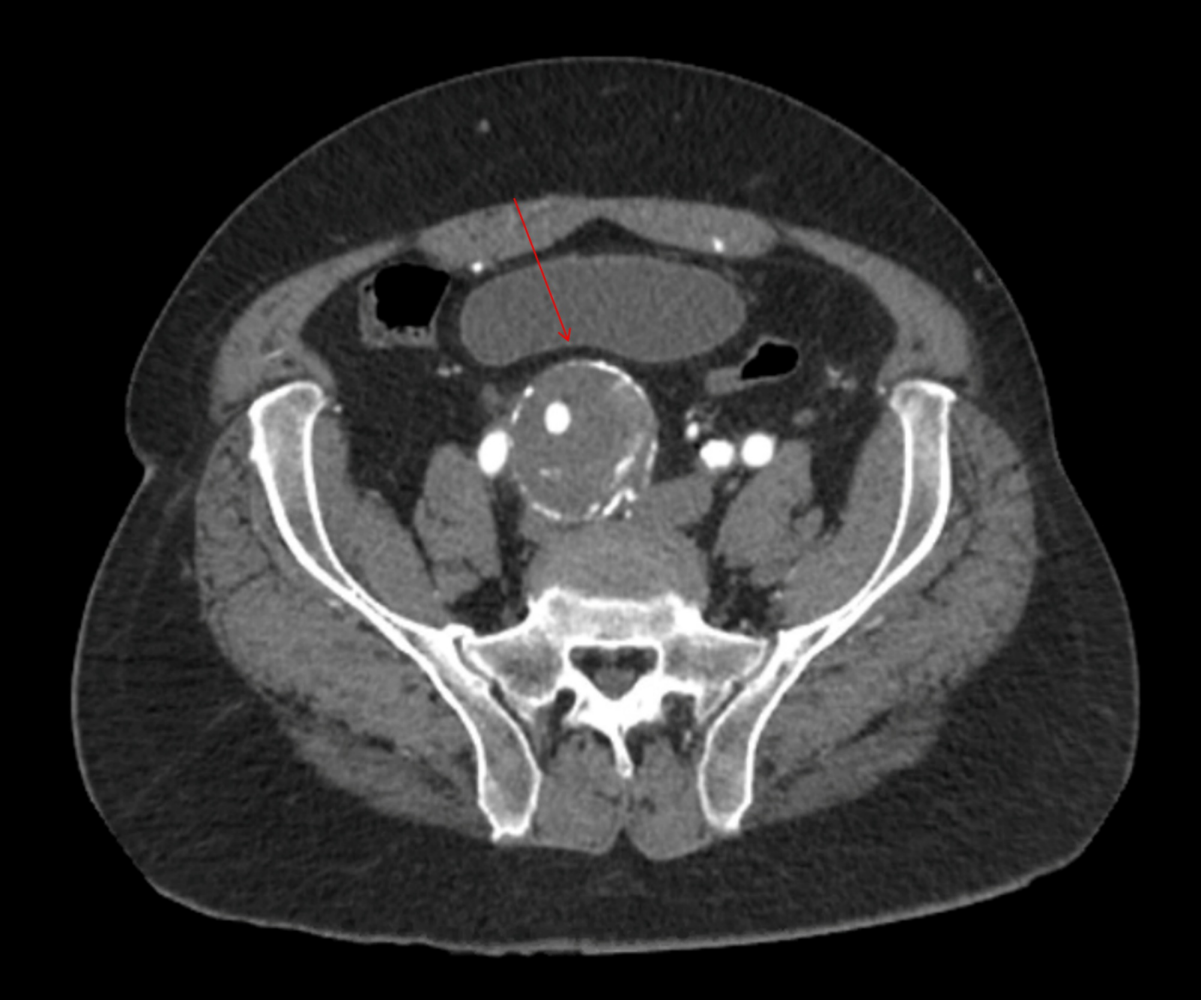
Lower in the pelvis, after bifurcation of both common iliac arteries, the internal iliac artery aneurysm visibly abutting the spine at the level of iliac crest

Reported five-year rupture rates for IAA range from 14% to 70%, and as many as 33% of patients with isolated iliac aneurysms present with rupture [5-7]. Rapid aneurysmal expansion (i.e., ≥7 mm in six months or >10 mm in one year) increases the risk of IAA rupture and indicates the need for repair. The internal iliac artery (or hypogastric artery), which is the smaller terminal branch of the common iliac artery, supplies the pelvic walls, pelvic viscera, external genitalia, perineum, buttock, and medial part of the thigh. Rupture can cause unilateral buttock claudication, which may occur in 16% to 50% of cases and bilaterally in up to 80%. As seen with our patient, due to the location being deeper in the pelvis, IIAAs have the tendency to silently enlarge and cause symptoms only once they cause mass effect, pressing on neighboring structures (Figure 7). Sexual dysfunction can also occur in 10% to 17% of cases, and uncommonly, spinal cord injury, ischemic colitis, and gluteal muscle necrosis can occur.

The planning of surgical treatment for multiple aortic aneurysms is influenced by location, diameter, and signs/symptoms. For patients who do not have signs or symptoms, elective repair should be planned. For those who are symptomatic and have a high risk of rupture, repair should be performed urgently. Multiple aortic aneurysms can be safely managed, usually with staged operations. Long-term survival is possible.

Our case report demonstrates several interesting features. The diagnosis of a partially thrombosed 8-cm aneurysm of the internal iliac artery is very rare, and there are very few case reports related to this condition. Most patients with IIAA are asymptomatic and incidental imaging on presentation may reveal large sizes that have a high risk of rupture requiring urgent repair.

Simultaneous repair is performed for ascending and arch aneurysms, for thoracoabdominal and infra-renal aneurysms, and suprarenal and infra-renal abdominal aneurysms. The management of descending thoracic and infra-renal aortic aneurysms are individualized, and the diameter and symptoms of the aneurysms dictate the sequence of repair. More proximal aneurysms are usually treated first [2].

Elective surgical reconstruction is the treatment of choice. Similar to reconstruction, open surgical graft placement is a major procedure associated with procedure-related morbidity and mortality and has now been replaced with endovascular treatment, which is technically feasible and durable. Internal iliac artery flow preservation is technically challenging, and not possible in severe cases.

## Conclusions

Although IIAA is very rare, there should be a high index of suspicion in patients with significant smoking history, atherosclerosis, and hip pain. The most effective diagnostic modality is CT. However, the disadvantages of exposing the patient to radiation and intravenous contrast must be considered. Additional research is needed regarding screening and diagnostic techniques of IIAA to avoid asymptomatic expansion and the need for an urgent repair or patient mortality. Future prospective data collection, including surveillance of long-term mortality and morbidity rates in post-repair patients, as well as in high-risk patients (who are ineligible for repair), may greatly expand the body of knowledge on IIAA.
